# Glycogenes in Oncofetal Chondroitin Sulfate Biosynthesis are Differently Expressed and Correlated With Immune Response in Placenta and Colorectal Cancer

**DOI:** 10.3389/fcell.2021.763875

**Published:** 2021-12-13

**Authors:** Zi-Yi Wu, Yong-Qiao He, Tong-Min Wang, Da-Wei Yang, Dan-Hua Li, Chang-Mi Deng, Lian-Jing Cao, Jiang-Bo Zhang, Wen-Qiong Xue, Wei-Hua Jia

**Affiliations:** ^1^ State Key Laboratory of Oncology in South China, Collaborative Innovation Center for Cancer Medicine, Sun Yat-sen University Cancer Center, Guangzhou, China; ^2^ School of Public Health, Sun Yat-sen University, Guangzhou, China

**Keywords:** colorectal cancer, malaria, oncofetal chondroitin sulfate, TCGA, immune infiltration

## Abstract

Oncofetal chondroitin sulfate expression plays an important role in the development of tumors and the pathogenesis of malaria in pregnancy. However, the biosynthesis and functions of these chondroitin sulfates, particularly the tissue-specific regulation either in tumors or placenta, have not been fully elucidated. Here, by examining the glycogenes availability in chondroitin sulfate biosynthesis such as xylosytransferase, chondroitin synthase, sulfotransferase, and epimerase, the conserved or differential CS glycosylation in normal, colorectal cancer (CRC), and placenta tissue were predicted. We found that the expression of seven chondroitin sulfate biosynthetic enzymes, namely B4GALT7, B3GALT6, B3GAT3, CHSY3, CHSY1, CHPF, and CHPF2, were significantly increased, while four other enzymes (XYLT1, CHST7, CHST15, and UST) were decreased in the colon adenocarcinoma (COAD) and rectum adenocarcinoma (READ) patients. In the human placenta, where the distinct chondroitin sulfate is specifically bound with VAR2CSA on *Plasmodium* parasite-infected RBC, eight chondroitin sulfate biosynthesis enzymes (CSGALNACT1, CSGALNACT2, CHSY3, CHSY1, CHPF, DSE, CHST11, and CHST3) were significantly higher than the normal colon tissue. The similarly up-regulated chondroitin synthases (CHSY1, CHSY3, and CHPF) in both cancer tissue and human placenta indicate an important role of the proteoglycan CS chains length for *Plasmodium falciparum* VAR2CSA protein binding. Interestingly, twelve highly expressed chondroitin sulfate enzymes were significantly correlated to worse outcomes (prognosis) in both COAD and READ. Furthermore, we showed that the levels of chondroitin sulfate enzymes are significantly correlated with the expression of immuno-regulators and immune infiltration levels in CRCs and placenta, and involved in multiple essential pathways, such as extracellular matrix organization, epithelial-mesenchymal transition, and cell adhesion. Our study provides novel insights into the oncofetal chondroitin sulfate biosynthesis regulation and identifies promising targets and biomarkers of immunotherapy for CRC and malaria in pregnancy.

## Introduction

Glycosylation is the most frequent post-translational modification occurring in proteins and lipids, and is vital to cell adhesion and stability as well as cell-cell communication (Dennis et al., 1999). Chondroitin Sulfate (CS) is one of the common glycosylation types with a glycosaminoglycan (GAG) linked to the backbones of specific proteoglycans (Afratis et al., 2012), which can vary greatly in length or degree of sulfation (Esko et al., 2009). Oncofetal CS (ofCS), the key player in malaria in pregnancy, normally restricted to trophoblastic cells in the placenta, has recently been detected in plenty of tumors. Importantly, the unprecedented high binding specificity and affinity of ofCS to *Plasmodium falciparum* VAR2CSA protein, which has been evolutionarily selected for use in placenta-bound parasites (Deitsch and Dzikowski, 2017; Ma et al., 2021), allows for a broad targeting of human cancer cells and placenta (Salanti et al., 2015). Understanding how ofCS is regulated in cancer tissue or placenta, therefore, is critical for tumor biology as well the pathogenesis of malaria in pregnancy.

**FIGURE 1:**
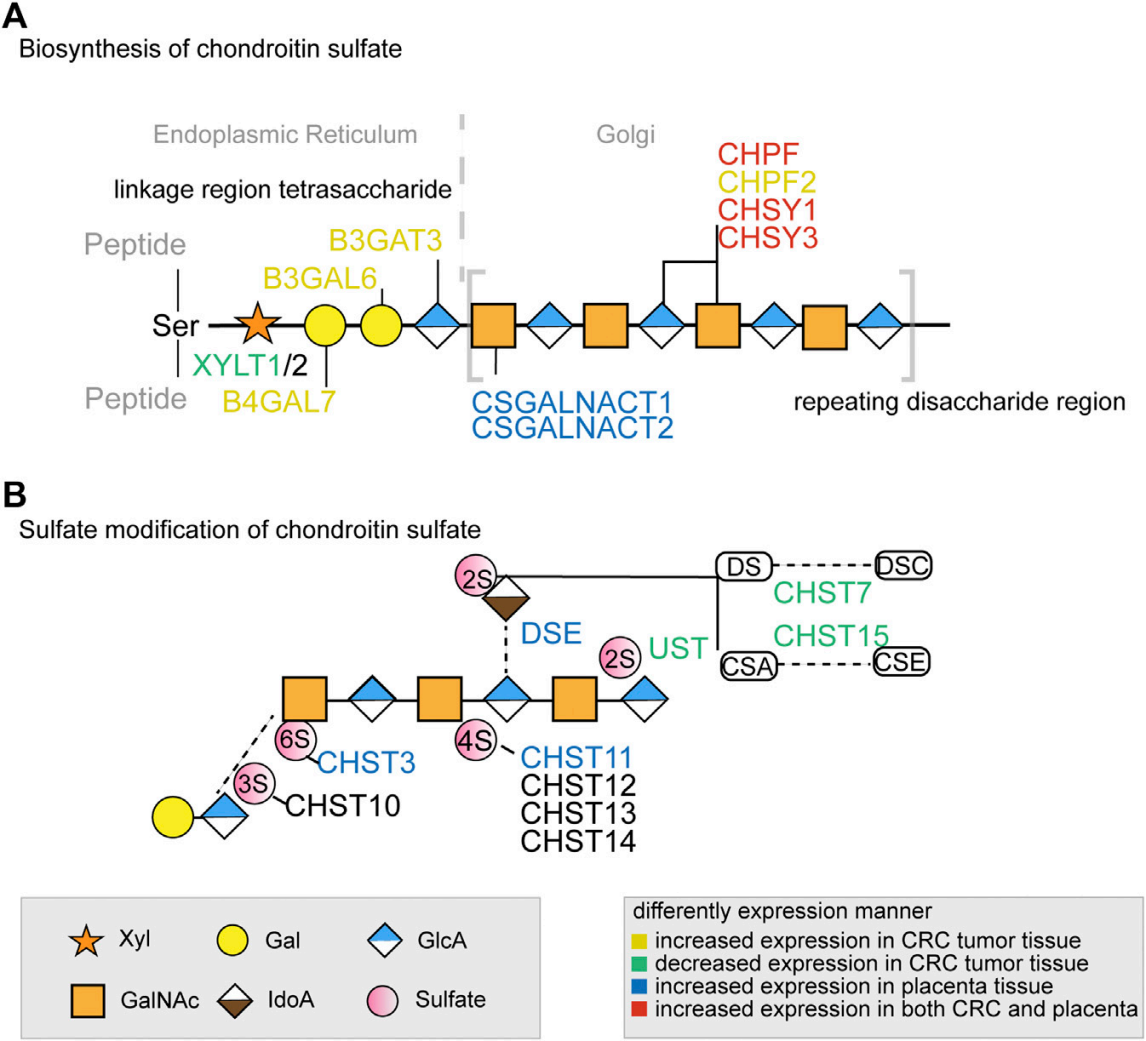
The key glycogenes in the biosynthesis of the common tetrasaccharide linkage and repeating disaccharide region of CS **(A)** and the glycogenes involved in sulfate modification **(B)** in colorectal cancer and placenta. The glycogenes in yellow represented those with increased expression in CRC tumor tissue, in green represented those with decreased expression in CRC tumor tissue, in blue represented those with increased expression in placenta tissue, and in red represented increased expression in both CRC and placenta. All monosaccharide symbols were followed using the Symbol Nomenclature for Glycans (SNFG) (https://www.ncbi.nlm.nih.gov/glycans/snfg.html).

Through 16 distinct glycosylation pathways, cellular glycan biosynthesis in human is facilitated by ∼500 glycoenzymes including glycosyltransferases, glycosidases, sugar metabolism enzymes, sulfation-related enzymes, and others (Neelamegham and Mahal, 2016; Schjoldager et al., 2020), which lead to the formation of complex and specific glycan structures on proteins or lipids (Potapenko et al., 2010). For example, glycosyltransferase catalyzes glycosidic bond formation between a sugar and an acceptor, forming a diverse range of saccharides and glycoconjugates in nature. According to the sequence homology, glycosyltransferases have been classified into different families in the Carbohydrate Active enzyme database (CAZy) with about 300 glycosyltransferases identified so far (Cazypedia, 2021). The assembly of linear or branched glycan chains is formed sequentially so that the product of an enzyme reaction prepares its acceptor as the substrate of the next enzyme in the process (Glavey et al., 2015). Of these glycoenzymes, a small minority are specifically involved in the CS biosynthesis pathway through the orchestration of enzymes’ kinetic properties and their compartmentalization in Golgi. In brief, the common tetrasaccharide linker of CS chain is initiated by xylosyltransferase1 (XYLT1) and XYLT2 and elongated by galactosyltransferases and glucuronyltransferase (Figure 1). After the linker assembly, two homologous *β*4GalNAc-transferases, encoded by Csgalnact1 and Csgalnact2, initiate CS synthesis, while chondroitin synthase and polymerizing enzyme, encoded by four homologous genes Chsy1, Chsy3, Chpf, and Chpf2, are responsible for the elongation of the GalNAc-*β* 1-4GlcA-*β* 1-3 repeat backbone which determines the length of CS chains (Mikami and Kitagawa, 2013; Chen et al., 2018). Lastly, the sulfation modification of CS is carried out by various sulfotransferase which catalyzes the transfer of a sulfate group to their respective sulfation sites on GalNAc, GlcA, or IdoA residues in CS chains (Mikami and Kitagawa, 2013).

Aberrant glycosylation, which often modulates inflammatory responses, enables host-pathogen interactions and immune escape, promotes cancer cell metastasis, or regulates apoptosis, has been linked with various diseases (Reily et al., 2019). For example, the abnormal expression of glycan has been linked with various cancers, including colorectal cancer (CRC, colon, and rectal cancer), one of the most common gastrointestinal tumors (Siegel et al., 2020). In malaria, the carbohydrate-mediated glycobiology can directly affect *Plasmodium* survival or host resistance (Gomes et al., 2017). The early study by Miller et al. showed the glycan on the erythrocyte surface mediate the parasite-red blood cell invasion, and the associated molecular was confirmed subsequently as glycophorins (Miller et al., 1977; Salinas et al., 2014). Interestingly, chondroitin sulfate A (CSA) in the human placenta, which mediates the binding of the infected RBC, was recently found uniquely expressed on the cancer cell surface and plays an important role in integrin signaling and tumor cell motility (Clausen et al., 2016). It appears that fine-tuned machinery for chondroitin sulfate biosynthesis is crucial for the CS chain expression in developmental and pathophysiological processes (Mikami and Kitagawa, 2013). However, the dynamic process of glycoconjugate synthesis, which depends on the local milieu of enzymes, sugar precursors, and organelle structures as well as the cell types and cellular signals involved (Reily et al., 2019), poses huge challenges for fully understanding the function and biosynthesis of ofCS in cancer or placenta. Recent studies suggest that glycan biosynthesis can be analyzed based on “gene availability” through the measure of the expression (proteome and transcriptome), accessibility (epigenome), or presence (genome) of glycogenes. Gene expression, either absolute or differential expression, defines which reactions could be active in the biosynthetic network and therefore could be used to predict differential glycosylation (Kellman and Lewis, 2021).

Herein, we systematically analyzed the glycogenes availability by comparing the expression of chondroitin sulfate biosynthesis enzymes from hundreds of CRC samples using The Cancer Genome Atlas (TCGA) RNA sequencing data and placenta transcriptomic database GSE66622. We found that three chondroitin synthases (CHSY1, CHSY3, and CHPF) were upregulated in both cancer tissue and placenta, indicating an important role of ofCS chains length for VAR2CSA binding. In addition, the differentially expressed chondroitin sulfate genes were significantly correlated to the prognosis for both COAD and READ, possibly due to their close association with the immune cell infiltration and the immune regulators.

## Materials and Methods

### Study Population and Data Collection

Colon adenocarcinoma (COAD) and rectum adenocarcinoma (READ) RNA sequence datasets, available from public databases, were used in this paper. The placenta RNA sequencing data from the GSE66622 and “HPA RNA-seq normal tissues” project were downloaded for comparison (Fagerberg et al., 2014; Hughes et al., 2015). For the RNAseq dataset, fragment per kilobase million (FPKM) values and reads per kilobase of exon per million reads mapped (RPKM) values were downloaded and converted to TPM (transcripts per kilobase of exon model per million mapped reads) and log2 transformed after the addition of 1 [log2 (x + 1)] before analysis (Goldman et al., 2020). Clinical annotations, including age, gender, histologic type, and clinical stages were acquired using the Cancer Genomics Data Server package (cgdsr) in the R environment (Cerami et al., 2012; Gao et al., 2013). Recently annotated glycogenes in the chondroitin sulfate biosynthesis pathway (Schjoldager et al., 2020) were compared, and gene names and functions are summarized in Supplementary Table S2. CNV and mutation data were obtained from the online genomic database cBioPortal (Cerami et al., 2012). The stemness score of individual samples was calculated using the methylation data and mRNA data as reported (Hoadley et al., 2018), and the data generated from the UCSC Xena dataset were used for correlation analysis (Goldman et al., 2020).

### Survival Analysis

Overall survival (OS) was defined by the time from diagnosis until death (patients alive at last follow-up were censored). For each gene, the optimal cutoff of high and low expression levels was determined using the surv-cutpoint function in the “survminer” R package (https://CRAN.Rproject.org/package=survminer). Kaplan-Meier survival curves were used to plot high and low expression groups for prognostic genes. Groups were compared using the Log-Rank test.

### Immune Cell Infiltration and Immune Regulators Correlation

According to the survival analysis, the prognosis-related genes were identified. The genes related to each immune cell infiltration level in tumor tissues were detected using the TIMER tool, which is a previously published statistical method to infer the abundance of tumor-infiltrating immune cells (TIICs) from gene expression profiles (Li et al., 2016; Li et al., 2017). The immune regulators were obtained from the TISIDB (Ru et al., 2019), and each prognosis-associated gene was performed for the correlation results. As not all the immune regulators were expressed in the fetus stage, we only analyzed the available regulators in this study. Heatmaps boxplots and a bubble diagram were generated in R using pheatmap (https://CRAN.R-project.org/package=pheatmap), ggplot2 (https://CRAN.R-project.org/package=ggplot2), and ggpubr (https://CRAN.R-project.org/package=ggpubr) packages.

### Enrichment Pathway Analysis

The top 100 co-expression genes of the chondroitin sulfate regulators were obtained from the Gene Expression Profiling Interactive Analysis (GEPIA; http://gepia2.cancer-pku.cn) databases (Tang et al., 2017; Tang et al., 2019). In this study, enriched pathways from co-expression of prognosis-associated genes were identified by Metascape (http://metascape.org) (Zhou et al., 2019), a user-friendly online analysis tool for common and unique pathways analysis. The top 20 pathways were visualized in R using ggplot2 (https://CRAN.R-project.org/package=ggplot2).

### Statistical Analysis

For data analysis, we used the statistical programming language R version 3.6.0 (R: A Language and Environment for Statistical Computing, R Core Team, R foundation for Statistical Computing, Vienna, Austria, 2019, http://www.R-project.org) supplemented with specialized packages for statistical analyses and graphs. Population characteristics were described using medians and percentages. Wilcoxon’s signed-rank test was used to compare matched normal and tumor tissues in the TCGA dataset. Pearson’s test was used for gene correlation analysis. Differences were considered to be significant if *p* < 0.05 after correction for multiple testing.

## Results

### Population Characteristics of the Individuals Enrolled in This Study

The baseline characteristics of the colorectal cancer patients enrolled in this study were summarized in Supplementary Table S1. Population characteristics in our cohort included 324 colon adenocarcinoma (COAD, including 41 normal adjacent tissue samples) patients and 102 rectum adenocarcinoma (READ, including 10 normal adjacent tissue samples) patients who had initial pathologic diagnosis at a median age of 64.9 years and 62.9 years, respectively. There were 154 (54.4%) males in the COAD cohort and 60 (54.3%) males in the READ cohort. Most tumors were adenocarcinoma, 244 (86.2%) and 85 (92.4) for COAD and READ respectively. More than half of patients did not have first-degree relatives with a cancer diagnosis. For placenta data, five normal colon samples and four placenta tissue RNA sequencing data were downloaded from the “HPA RNA-seq normal tissues” project, and 80 placenta samples from GSE66622 were included.

### Differentially Expressed Glycogenes in Chondroitin Sulfate Biosynthesis in Colorectal Cancer and Placenta

**FIGURE 2:**
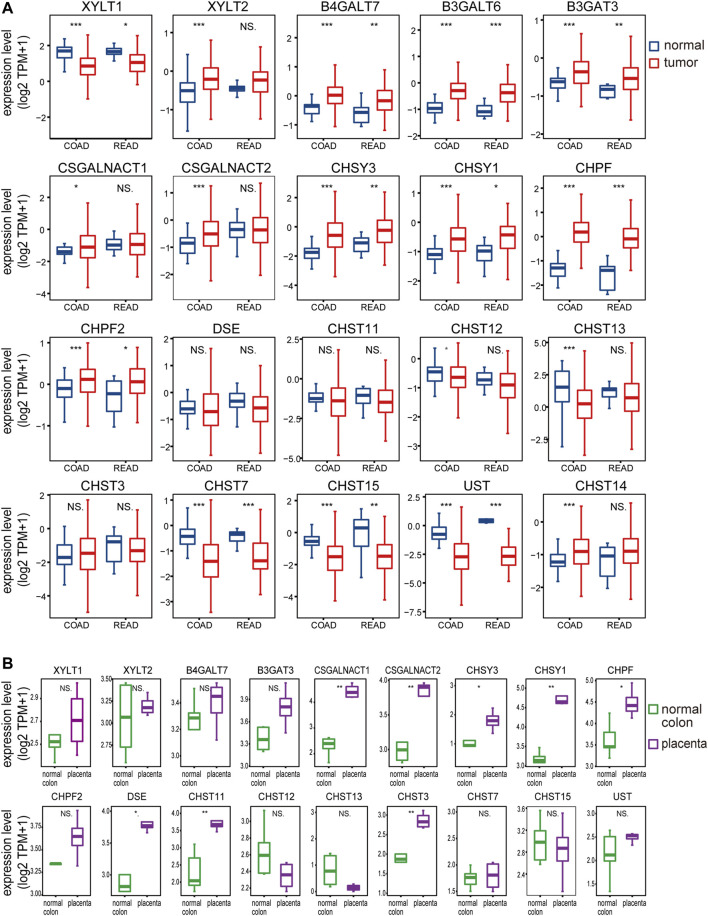
The chondroitin sulfate glycogenes are differentially expressed in tumors and placenta. The expression profile of chondroitin sulfate biosynthesis glycogenes in normal and tumor of colon and rectal **(A)** and normal colon vs placenta **(B)**. Transcripts per million transcripts (TPM) values in each chondroitin sulfate glycogenes were log2 transformed after the addition of 1 [log2 (x + 1)] and visualized as a boxplot.

Previous studies of chondroitin sulfate biosynthesis genes expression were only performed in cancer with small sample sizes and limited data coverage (Bret et al., 2009). To provide a more comprehensive evaluation of ofCS biosynthesis, we compared mRNA expression in both placenta (Fagerberg et al., 2014; Hughes et al., 2015) and colorectal cancer (UCSC TOIL, (Vivian et al., 2017). In COAD and READ patients, we noticed that galactosyltransferases (B4GALT7, B3GALT6), glucuronyltransferase (B3GAT3), chondroitin synthase (CHSY3, CHSY1, CHPF2), and polymerizing enzyme (CHPF) were significantly increased compared to the normal adjacent tissues, while xylosyltransferase (XYLT1) and ofCS modification enzymes (CHST7, CHST15, UST) were both decreased. Other ofCS modification enzymes (DSE, CHST11, and CHST13) were not differentially expressed between normal and tumor samples (Figure 2A). Importantly, there were 17 out of the 20 genes differentially expressed in the COAD patients, suggesting an important role for regulation in determining chondroitin sulfate synthesis and its functional status in colon cancer. Further analysis indicated that XYLT1, XYLT2, B4GALT7, B3GALT6, B3GAT3, CSGALNACT2, CHSY3, CHSY1, CHPF, CHST13, CHST7, CHST15, UST, and CHST14 were up/down-regulated in all clinical stages of COAD (Supplementary Figure S1). Meanwhile, B4GALT7, B3GALT6, CHPF, and CHST15 were up/down-regulated in all stages of READ patients (Supplementary Figure S2).

In the placenta, there were eight chondroitin sulfate biosynthesis enzymes (CSGALNACT1, CSGALNACT2, CHSY3, CHSY1, CHPF, DSE, CHST11, and CHST3) that were significantly higher than the normal colon tissue (Figure 2B), while B3GALT6 and CHST14 were not available from the dataset. Three chondroitin synthases (CHSY1, CHSY3, and CHPF) were upregulated in both cancer and placenta, indicating the length of ofCS chains might be an important factor for VAR2CSA binding. A detailed comparison of glycogenes expression changes in the placenta and cancer was illustrated in Figure 1.

### Amplification, Deletion, and Mutation of Chondroitin Sulfate Biosynthesis Regulators in Colorectal Cancer

**FIGURE 3:**
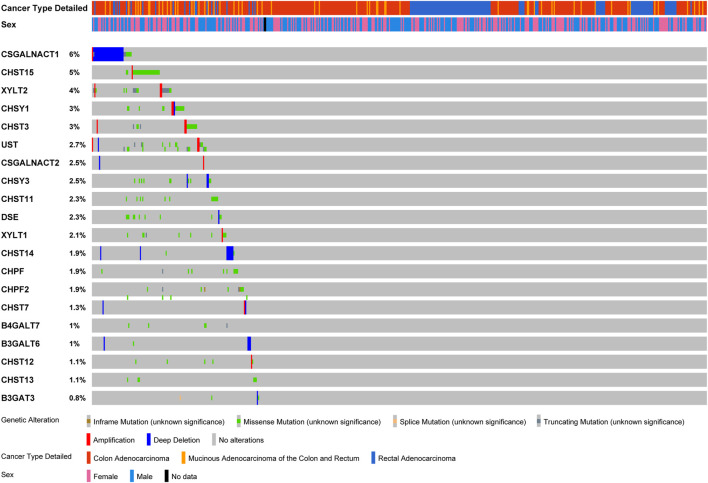
Landscape of chondroitin sulfate biosynthesis glycogenes alteration in colorectal cancer. Genetic alterations of chondroitin sulfate biosynthesis glycogenes (originated from TCGA studies) were detected using the cBioPortal database. The amplification, deep deletion, truncating mutation, and missense mutation were shown in different colors in each glycogene.

The variations of glycogenes in chondroitin sulfate biosynthesis in COAD and READ were further explored using the cBioPortal database (Figure 3). We found varying degrees of genetic changes among the 20 chondroitin sulfate biosynthesis genes, consistent with previous reports (Hansen et al., 2015; Joshi et al., 2018). As shown in Figure 3, we revealed that most of the chondroitin sulfate biosynthesis glycogenes were deleted, missense mutated, or amplified in CRC, among which CSGALNACT1 displayed the highest incidence rate (6%), followed by CHST15 (with an incidence rate of 5%).

### Gene Expression in Relation to Overall Survival

**FIGURE 4:**
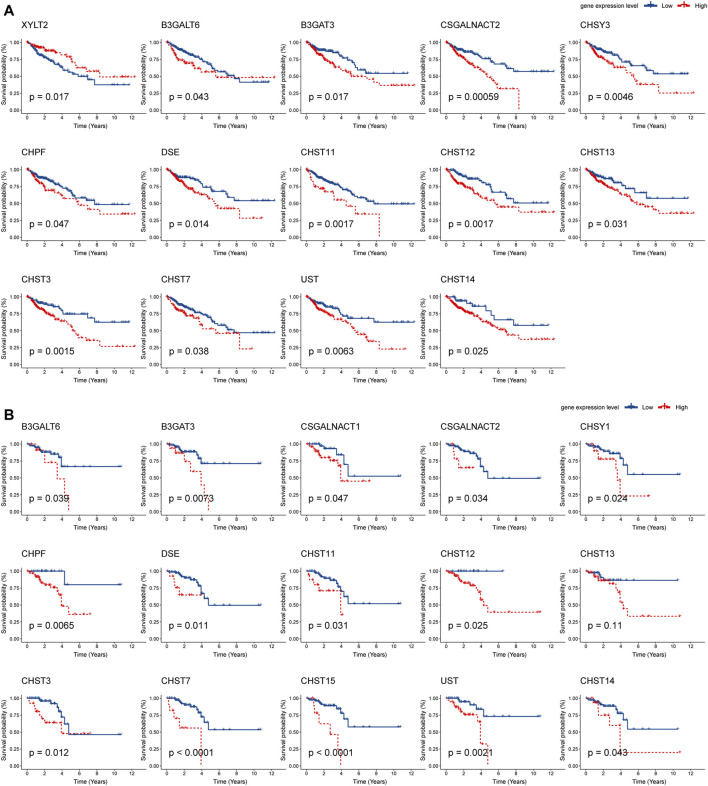
Prognostic analysis of chondroitin sulfate biosynthesis glycogenes in colorectal cancer. The correlation between chondroitin sulfate biosynthesis glycogenes and overall survival (OS) in COAD **(A)** and READ **(B)** is displayed. The red line denotes higher expression, and the blue line indicates lower expression.

Next, Kaplan–Meier plot was used to analyze the correlation between the mRNA levels of chondroitin sulfate biosynthesis enzymes and CRC patient prognosis. Interestingly, high expression of most glycogenes was significantly associated with shorter overall survival (OS) rate in COAD and READ. These genes include B3GALT6, B3GAT3, CSGALNACT2, CHPF, DSE, CHST11, CHST12, CHST13, CHST3, CHST7, UST, and CHST14, indicating that chondroitin sulfate biosynthesis enzymes play key roles in colorectal cancer (Figure 4).

### The Expression of Prognosis Associated Chondroitin Sulfate Biosynthesis Genes is Correlated With Immune Infiltration Levels and Stemness in Colorectal Cancer and Placenta

**FIGURE 5:**
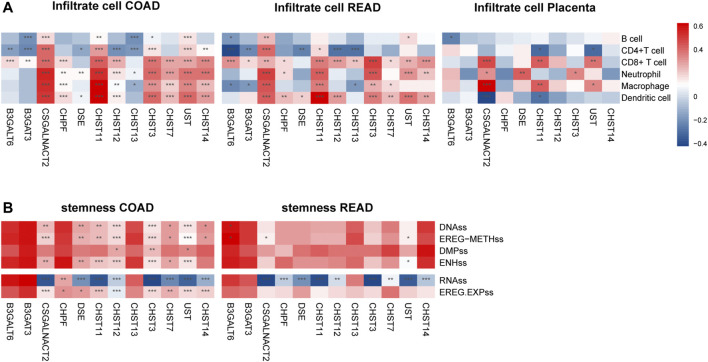
The expressions of twelve chondroitin sulfate biosynthesis glycogenes are correlated with immune infiltration levels **(A)** and stemness **(B)** in COAD, READ, and placenta. *p < 0.05, **p < 0.01, ***p < 0.001. Genes with the higher correlation of the immune infiltration levels are shown in red, while those with lower levels are shown in blue.

The survival time of many cancers is determined by the amount and activity status of TILs (tumor-infiltrating lymphocytes) (Ohtani, 2007; Santoiemma and Powell, 2015). Based on the TIMER estimate method, we detected the correlation of 12 prognosis-associated chondroitin sulfate biosynthesis genes with levels of immune cell infiltration in COAD and READ. As present in Figure 5A, much of the gene expression was positively associated with the level of immune cells. For example, CSGALNACT2 expression was associated with level of dendritic cell (*r* = 0.61, *p* = 1.06E-42), neutrophil (*r* = 0.64, *p* = 2.13E-47), macrophage (*r* = 0.66, *p* = 8.5E-53), CD4^+^ T Cell (*r* = 0.49, *p* = 6.08E-26), and CD8^+^ T Cell (*r* = 0.48, *p* = 5.05E-25). Similarly, CHST11 was associated with the level of dendritic cell (*r* = 0.74, *p* = 7.35E-70), neutrophil (*r* = 0.73, *p* = 1.03E-66), macrophage (*r* = 0.64, *p* = 4.69E-48), CD4^+^ T Cell (r = 0.51, *p* = 2.26E-28), and CD8^+^ T Cell (*r* = 0.38, *p* = 3.01E-15). In addition, CHST3, CHST7, UST, and CHST14 were all significantly associated with dendritic cell, neutrophil, macrophage, and CD4^+^ T Cell infiltration.

For the READ dataset, similar trends were detected between the prognosis associated genes expression with the immune cell infiltration. CSGALNACT2 was associated with the increased dendritic cell (*r* = 0.43, *p* = 1.47E-7), neutrophil (*r* = 0.46, *p* = 2.06E-8), macrophage (*r* = 0.49, *p* = 7.24E-10), CD4^+^ T Cell (*r* = 0.27, *p* = 1.55E-3), and CD8^+^ T Cell (*r* = 0.40, *p* = 9.03E-7). CHST11 was associated with increased dendritic cell (*r* = 0.63, *p* = 1.86E-16), neutrophil (*r* = 0.35, *p* = 2.11E-5), macrophage (*r* = 0.43, *p* = 1.25E-7), and CD4^+^ T Cell (*r* = 0.36, *p* = 1.13E-5).

Interestingly, in placenta tissue, CSGALNACT2 was associated with increased level of macrophage (*r* = 0.52, *p* = 7.44E-07), neutrophil (*r* = 0.27, *p* = 0.015), and CD8^+^ T cell (*r* = 0.38, *p* = 4.53E-4), while it was negatively associated with dendritic cell (*r* = −0.36, *p* = 8.77E-4). CHST11 was also significantly associated with macrophage and CD8^+^ T cell (*r* = 0.35, *p* = 1.40E-3, *r* = 0.35, *p* = 1.60E-3, respectively) (Figure 5A).

Cancer stemness was thought to be accountable for resistance to anti-cancer therapies and subsequent relapse. To explore the correlation of the prognosis associated genes expression with the stemness of the same sample, we utilized the stemness score determined by the DNA hypermethylation data and mRNA (Hoadley et al., 2018). As shown in Figure 5B, CAGALNACT2, DSE, CHST11M CHST12 CHST3, CHST7, UST, and CHST14 in the COAD were positively associated with the DNAss category, EREG-METHs category, ENHss category, which was derived from the methylation data, and EREG EXPss, derived from the mRNA data. However, they were negatively associated with the RNAss category derived from the mRNA data. There were significantly negative associations between the gene expression of CHPF, DSE, CHST11, CHST12, CHST3, UST CHST14, and stemness of RNAss category for READ.

### Correlations Between the Expression of Prognosis Associated Chondroitin Sulfate Biosynthesis Enzymes and Immuno-Regulators in Colorectal Cancer and Placenta

**FIGURE 6:**
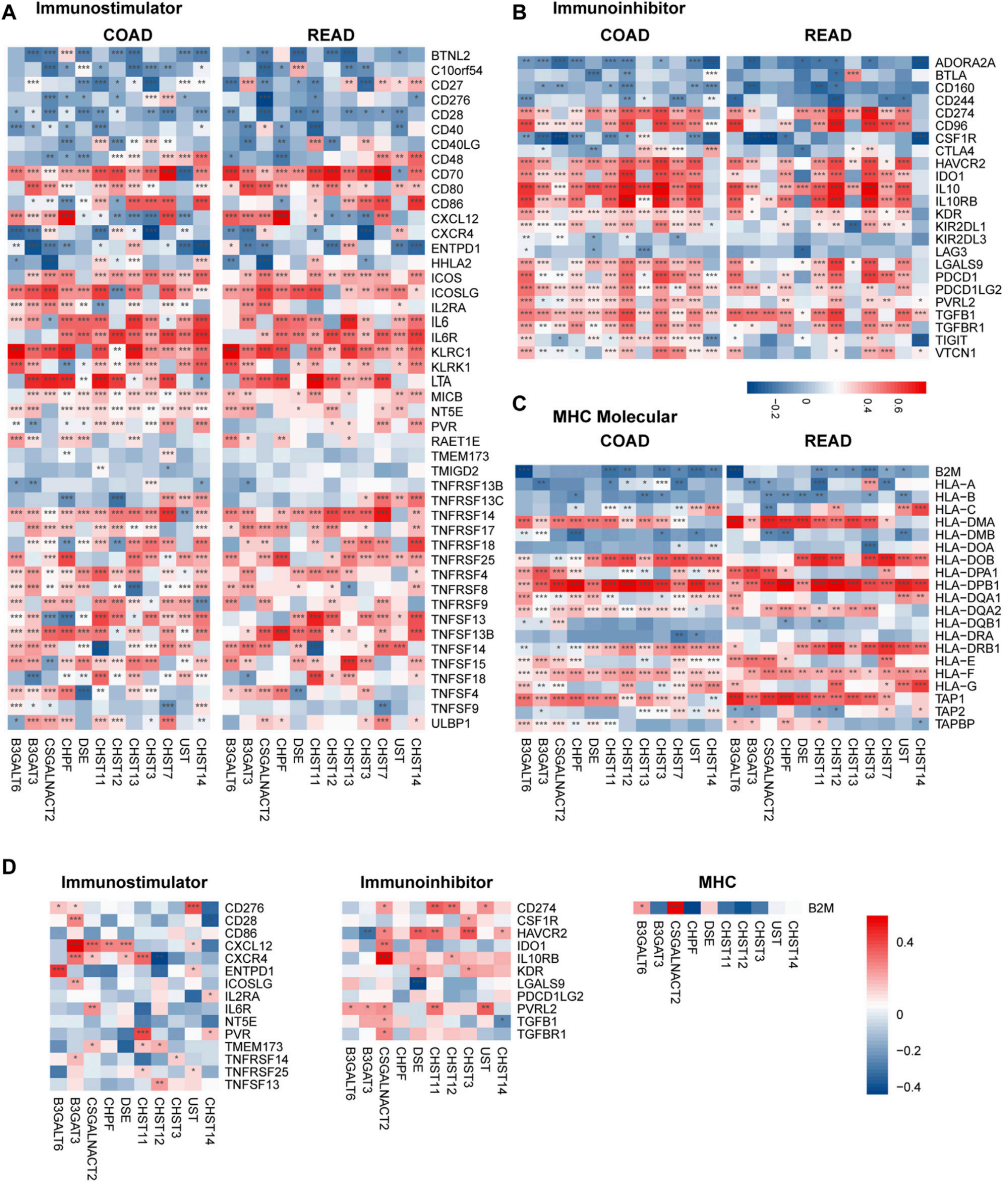
Correlations between chondroitin sulfate regulators expression and the expression of Immuno-regulators in COAD, READ, and placenta tissue. The correlations between the expression of 12 progress-associated chondroitin sulfate biosynthesis regulators and Immunostimulator **(A)**, Immunoinhibitors **(B)**, and MHC molecules **(C)** for CRC tissue and the placenta tissue **(D)**. Genes with the higher correlation of the immune regulator’s levels are shown in red, while those with lower levels are shown in blue. *p < 0.05, **p < 0.01, ***p < 0.001.

To further explore the effects of chondroitin sulfate biosynthesis genes on immune response in tumor and placenta, the correlations between the expression of prognosis associated chondroitin sulfate biosynthesis glycogenes and immuno-regulators were calculated (Charoentong et al., 2017). The results indicated that many of these genes were significantly correlated to immunostimulators (Figure 6A), immunoinhibitors (Figure 6B), and MHC molecules (Figure 6C) in cancer and placenta (Figure 6D).

### GO and KEGG Enrichments Analysis

**FIGURE 7:**
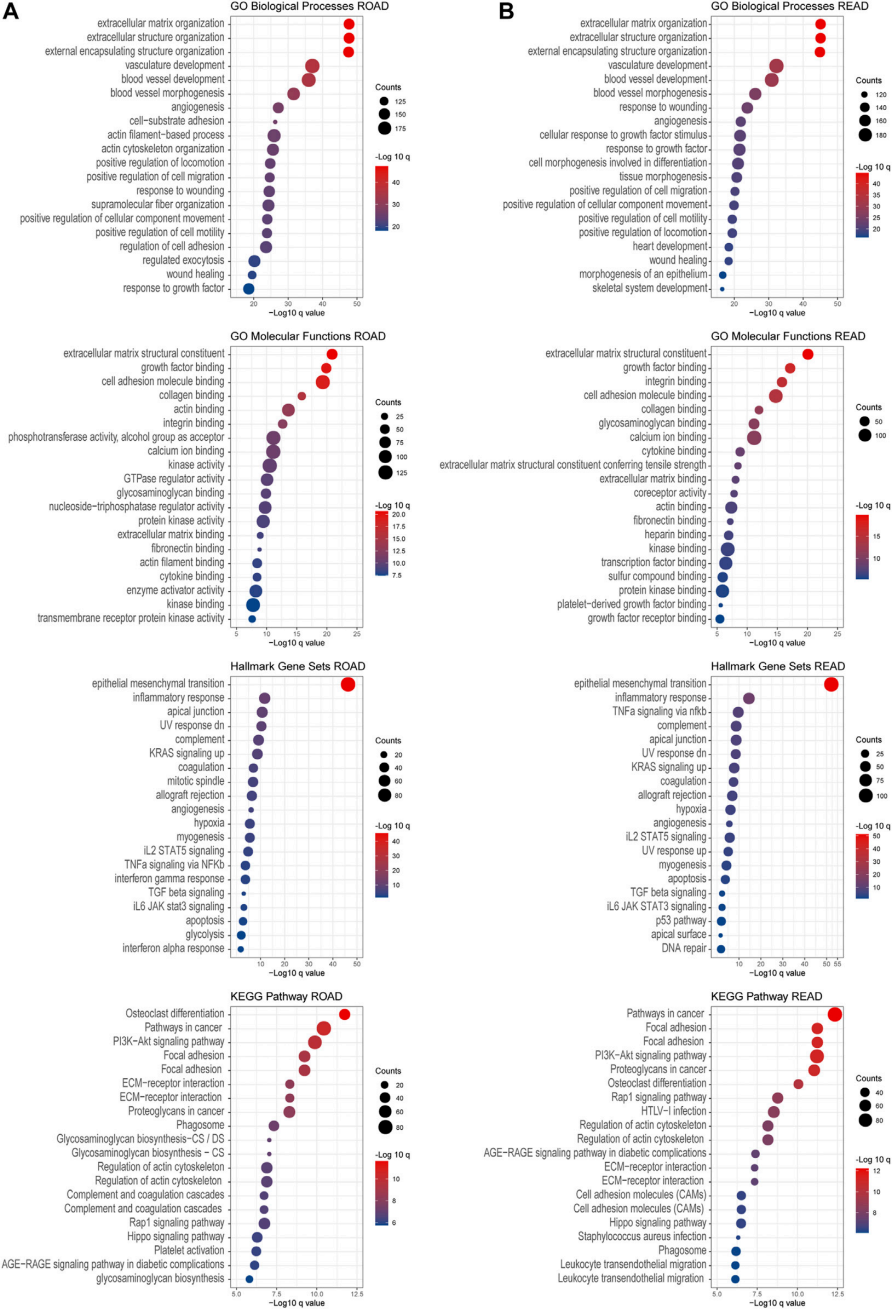
GO and KEGG enrichment analysis. Gene Ontology enrichment of the co-expressed genes associated with chondroitin sulfate glycogenes was identified through gepia2. Analysis of the “GO biological process” category, “GO molecular functions” category, “Hall mark gene sets” category, and “KEGG pathway category” for COAD dataset **(A)** and READ dataset **(B)**. The color of the dots demonstrates -Log10 (*q* value). Therefore, red dots show greater FDR than blue ones. The size of the dots indicates the number of genes enriched in the analysis.

To investigate the downstream pathways of prognosis associated with chondroitin sulfate biosynthesis glycogenes in CRC, we performed GO and KEGG analysis using co-expression genes. The results indicated that the co-expression molecular of COAD were enriched in the extracellular matrix organization, extracellular matrix structural constituent, which was correlated with cell adhesion molecule binding. The enrichment of the pathways in the epithelial-mesenchymal transition process and KRAS signaling up shows that PI3K-Akt signaling supports the critical role of these genes in cancer development (Figure 7A). The co-expression molecular was similar in the COAD and READ, as the enrichment signals revealed the similarities in performance (Figure 7B).

**FIGURE 8:**
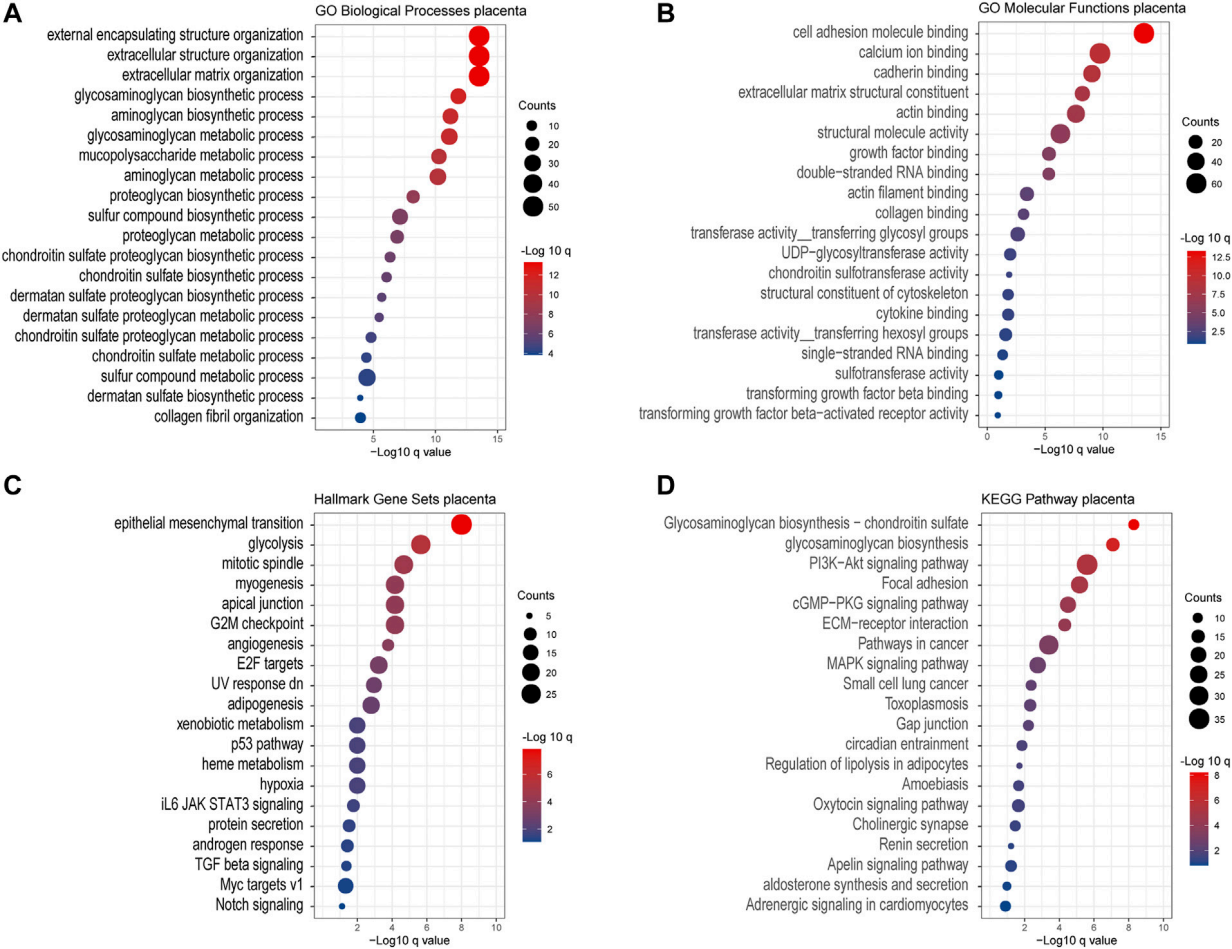
Gene Ontology enrichment of the co-expressed genes associated with chondroitin sulfate regulator glycogenes in the placenta. Analysis of the “GO biological process” category **(A)**, “GO molecular functions” category **(B)**, “Hall mark gene sets” category **(C)**, and “KEGG pathway category” **(D)** for the placenta. The color of the dots demonstrates -Log10 (*q* value). Therefore, red dots show greater FDR than blue ones. The size of the dots indicates the number of genes enriched in the analysis.

Except for the glycan biosynthesis process, enrichment analysis of co-expressed genes in the placenta (Figure 8) showed similar processes as in cancer, such as extracellular matrix organization and cell adhesion molecular binding. Interestingly, we noticed that on the KEGG pathway category, the “pathway in cancers” was also significantly enriched.

## Discussion

Several studies investigated the role of chondroitin sulfate in cancer, providing evidence of their important roles in cell proliferation, migration, apoptosis, and differentiation (Keutgen et al., 2021; Sano et al., 2021). Glycosylation of proteins generates changes in their biophysical properties, function, distribution, and retention in the plasma membrane and modulates cell behavior, cellular interactions, specific ligand-receptor interactions, and immune recognition (Shental-Bechor and Levy, 2008; Vagin et al., 2009; Hoja-Łukowicz et al., 2013). Alterations in glycosyltransferase levels and glycosylation patterns have been evidenced in inflammatory conditions, tumorigenesis, and metastasis (Nardy et al., 2016). The measurements of glycosyltransferase expression at the mRNA level and direct enzyme expression are performed in most studies (Wang et al., 2005; Laidler et al., 2006; Trinchera et al., 2011; Glavey et al., 2015). Studies showed that alterations in the expression of some glycosylation-associated genes have been correlated with cancer progress and applied as cancer diagnostic biomarkers such as FUT8 (Taniguchi and Kizuka, 2015; Agrawal et al., 2017).

Until now, few studies have investigated the oncofetal chondroitin sulfate biosynthesis and the contribution of GAG structures to specific biological functions due partly to obstacles in technical limitation in the structural characterization. The development of a cell-based method, GAGome, enables the production and display of GAGs and interrogates the detailed structural features required for specific bioactivities (Chen et al., 2018). By precise gene editing (knock out or knock in of the glycogenes), the binding specificities of recombinant VAR2CSA (rVAR2CSA), fibroblast growth factor 2 (FGF2), and CS-56 monoclonal antibody to CS/HS were successfully determined. The *plasmodium falciparum* VAR2CSA protein is proposed to bind a distinct 4-O-sulfated CS that is expressed in the normal placenta (Ma et al., 2021) and most human cancers (Salanti et al., 2015). Three GAGOme sub-libraries were used to probe the key glycogenes of CS linker assembly, CS initiation/elongation, and CS modification for their effects on ofCS binding to rVAR2CSA. In this study, we investigated the transcriptional expression profile of all chondroitin sulfate biosynthesis enzymes in CRC and placenta by exploring COAD and READ, two expression datasets from TCGA, and the public normal tissue RNA sequencing data. The expression of the selected chondroitin sulfate biosynthesis genes discriminated between tumor and normal tissues. Seven consistent genes were increasingly expressed in COAD and READ tumor tissues compared to normal tissues, while four were decreasingly expressed in tumor tissues. More importantly, there were three genes, which encode the core glycoenzymes for CS chain elongation, that significantly increased in both colorectal cancer and placenta. The expression alteration of these glycosyltransferases could predict the impact of differential oncofetal chondroitin sulfate glycosylation on extracellular signaling in the placenta and tumor tissue.

In this study, we found that 12 high expressions of chondroitin sulfate biosynthesis glycogenes in the genome were correlated to a worse prognosis in human CRC. Some of these genes have been reported for their association with cancer prognosis. For example, the high expression of CHST11, the enzyme specifically required for CSA 4-O-sulfation, was significantly correlated with poor relapse-free survival in three independent lung cancer cohorts (Salanti et al., 2015). CSGALNACT1, which is necessary for normal cartilage development and aggrecan metabolism (Yoshioka et al., 2017), was related to integrin signaling and prognosis (Agnelli et al., 2011; Clausen et al., 2016). CHST15 was also critical for the prognosis in breast cancer and pancreatic cancer (Ito et al., 2017; Liu et al., 2019). Both CSGALNACT1 and CHST15 genes showed the highest incidence rate (5–6%) in deletion, mutation, and amplification in CRC. Further, GO and KEGG enrichment analyses revealed that co-expression prognosis associated with genes in the placenta and CRC mostly involved in “extracellular matrix organization”, “epithelial-mesenchymal transition”, and “cell adhesion”, supporting the shared mechanism in tumorigenesis and embryonic development in the placenta.

Immune components in the tumor microenvironment have essential effects on gene expression by tumor tissues and the clinical outcome. Cancer immunotherapy mainly works with some important proteins to enhance function or restore immune cells in the tumor microenvironment. For example, programmed death-1 (PD-1) on the surface of CD8^+^ T cells binds programmed death-ligand 1 (PD-L1) produced by tumor tissue, resulting in a limited host immune response (Tumeh et al., 2014). CD4^+^ T cells play an essential role in the formation of protective memory CD8 (+) T cells (Laidlaw et al., 2016). Macrophages and neutrophils are the primary effectors of the innate immune system potent immune effector cells to antitumors (Anderson et al., 2021). In this study, six different immune cells, namely B cell, CD8^+^ T cell, CD4^+^ T cell, macrophage, neutrophil, and dendritic cell, were explored to identify their correlation with the chondroitin sulfate glycogenes expression. Interestingly, the levels of CSGALNACT2 and CHST11 both showed a strong correlation with the infiltrate immune cells in COAD and READ patients and placenta. It has been reported that immunological changes in the placenta are associated with poor pregnancy outcomes in malaria infection (Sharma and Shukla, 2017). The link of key ofCS biosynthesis enzymes to the immune responses in this study provides possible new immunotherapy targets for malaria in pregnancy. Its role in placental immunopathology should be further explored in malaria infection.

It is worth noting that the differentially expressed glycogenes were identified by comparison of CRC tissue and placenta RNAseq data; the functions of these glycogenes still need further validation by precise genome-editing techniques such as CRISPR/Cas (Narimatsu et al., 2018; Narimatsu et al., 2021). Further, whether the abnormal glycosylation of tumor impacts immune therapy should be illustrated further (Chiang et al., 2021).

## Conclusion

In summary, we show that chondroitin sulfate regulators are differentially expressed in the CRC tumor site and placenta and increased expression of glycogenes predicts poor prognosis in CRC patients and could be considered as a biomarker or immune therapy targets for CRC. Our results provide novel insights about the chondroitin sulfate biosynthesis glycoenzymes in the placenta as well as CRC initiation and progression.

## Data Availability

The original contributions presented in the study are included in the article/Supplementary Material, further inquiries can be directed to the corresponding author.
