# Supplementary material for: Glycogenes in Oncofetal Chondroitin Sulfate Biosynthesis are Differently Expressed and Correlated With Immune Response in Placenta and Colorectal Cancer
Source: Front Cell Dev Biol. 2021 Dec 13;9:763875. doi: 10.3389/fcell.2021.763875 (PMC8710744; doi:10.3389/fcell.2021.763875)
Supplement: Supplementary file 1 [file DataSheet1.PDF]

## Supplementary Figures and Tables

# Glycogenes in Oncofetal Chondroitin Sulfate Biosynthesis are Differently Expressed and Correlated with Immune Response in Placenta and Colorectal Cancer

**Zi-Yi Wu, Yong-Qiao He, Tong-Min Wang, Da-Wei Yang, Dan-Hua Li, Chang-Mi Deng, Lian-Jing Cao, Jiang-Bo Zhang, Wen-Qiong Xue, Wei-Hua Jia,**

**Number of supplementary figures: 2**

**Number of supplementary tables: 2**

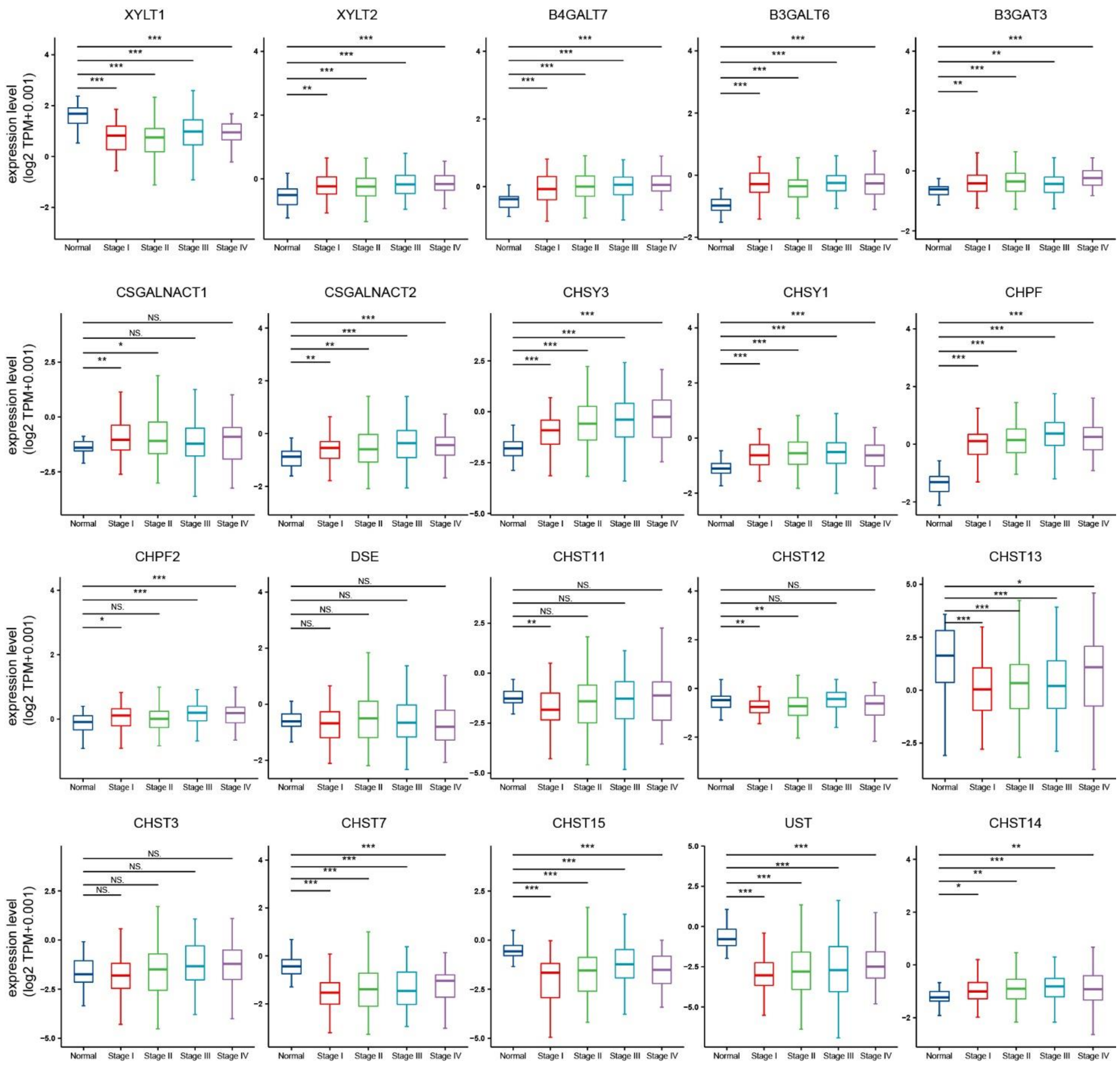


**Supplementary figure 1:** The dysregulation of chondroitin sulfate biosynthesis glycogenes are correlated to advanced clinical stage in COAD. The expression of XYLT1, XYLT2, B4GALT7, B3GALT6, B3GAT3, CSGALNACT1, CSGALNACT2, CHSY3, CHSY1, CHPF, CHPF2, DSE, CHST11, CHST12, CHST13, CHST3, CHST7, CHST15, UST and CHST14 were detected in colon adenocarcinoma.

NS. not significant, * $p < 0.05$, ** $p < 0.01$, *** $p < 0.001$.

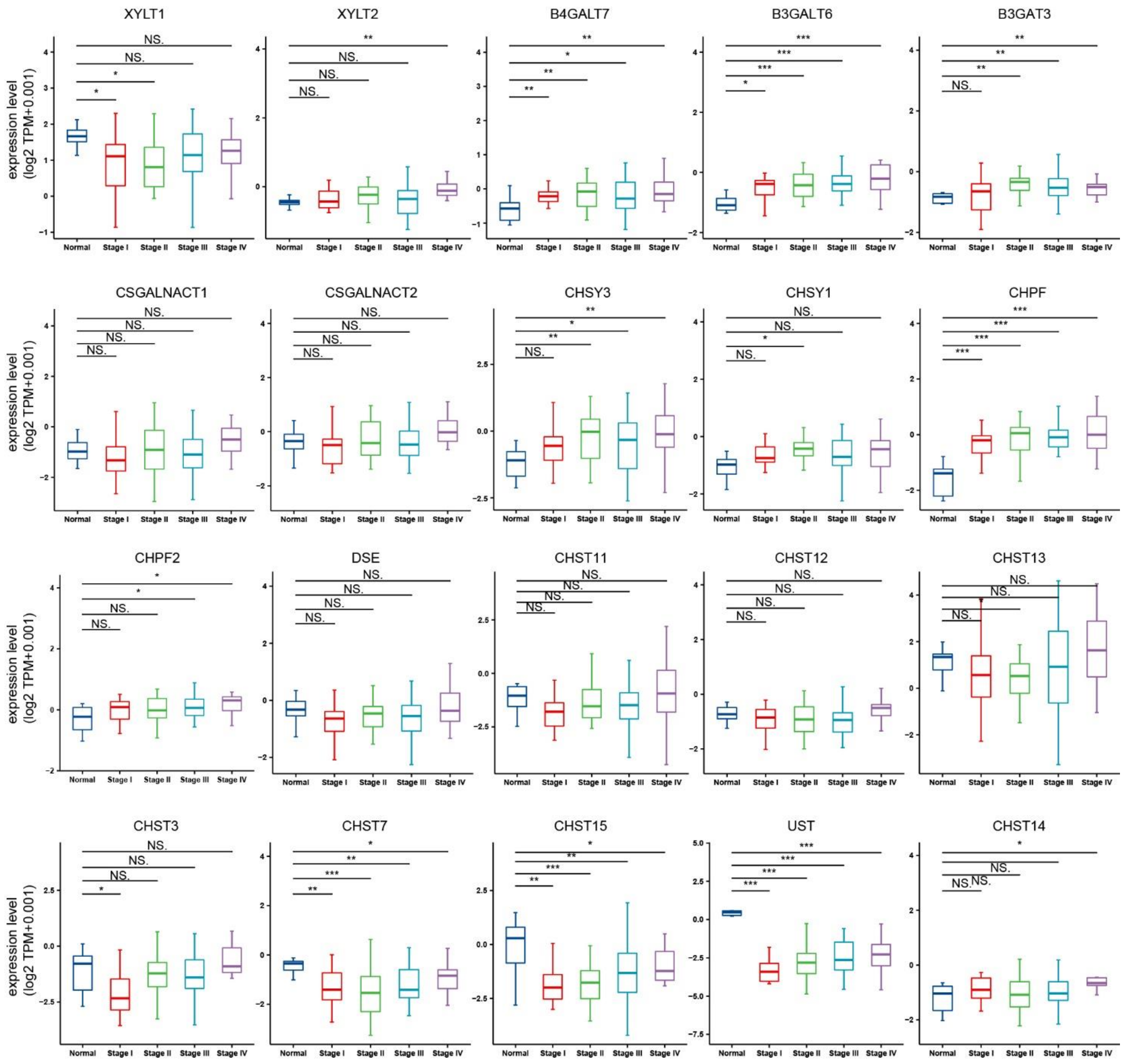


**Supplementary figure 2:** The dysregulation of chondroitin sulfate biosynthesis glycogenes are correlated to advanced clinical stage in READ. The expression of XYLT1, XYLT2, B4GALT7, B3GALT6, B3GAT3, CSGALNACT1, CSGALNACT2, CHSY3, CHSY1, CHPF, CHPF2, DSE, CHST11, CHST12, CHST13, CHST3, CHST7, CHST15, UST and CHST14 were detected in rectum adenocarcinoma.

NS. not significant, * $p < 0.05$, ** $p < 0.01$, *** $p < 0.001$.

**Supplementary Table 1 Basic characteristics of colorectal patients enrolled from TCGA database for analysis.**

| | COAD | | READ | |
|---|---|---|---|---|
| | **normal N=41(%)** | **tumor N=283(%)** | **normal N=10(%)** | **Tumor N=92(%)** |
| Average age, y (SD) | 70.3(13.2) | 64.9(13.2) | 63.8(16.9) | 62.9(12.6) |
| Gender | | | | |
| FEMALE | 21(51.2) | 129(45.6) | 7(70.0) | 42(45.7) |
| MALE | 20(48.8) | 154(54.4) | 3(30.0) | 50(54.3) |
| Histological type | | | | |
| Mucinous Adenocarcinoma | / | 36(12.7) | / | 5(5.43) |
| Adenocarcinoma | / | 244(86.2) | / | 85(92.4) |
| Unknown | / | 3(1.06) | / | 2(2.17) |
| Family history | | | | |
| No | 31(75.6) | 184(65.0) | 7(70.0) | 61(66.3) |
| Yes | 4(9.8) | 33(11,7) | 2(20.0) | 9(9.8) |
| Unknown | 6(14.6) | 66(23.3) | 1(10.0) | 22(23.9) |
| T stage | | | | |
| T1 | / | 6(2.12) | / | 4(4.35) |
| T2 | / | 44(15.5) | / | 13(14.1) |
| T3 | / | 194(68.6) | / | 63(68.5) |
| T4 | / | 39(13.8) | / | 10(10.9) |
| Unknown | / | / | / | 2(2.17) |
| N stage | | | | |
| N0 | / | 165(58.3) | / | 38(41.3) |
| N1 | / | 70(24.7) | / | 29(31.5) |
| N2 | / | 48(17.0) | / | 21(22.8) |
| Unknown | / | / | / | 4(4.35) |
| M stage | | | | |
| M0 | / | 189(66.8) | / | 63(68.5) |
| M1 | / | 39(13.8) | / | 12(13.0) |
| Unknown | / | 55(19.4) | / | 17(18.5) |
| AJCC stage | | | | |
| Stage I | / | 44(15.5) | / | 12(13.0) |
| Stage II | / | 110(38.9) | / | 24(26.1) |
| Stage III | / | 81(28.6) | / | 33(35.9) |
| Stage IV | / | 39(13.8) | / | 13(14.1) |
| Unknown | / | 9(3.18) | / | 10(10.9) |

**Abbreviations:** COAD: colon adenocarcinoma; READ: rectum adenocarcinoma

**Supplementary Table 2 Key glycogenes in the chondroitin sulfate biosynthesis pathway.**

| Entrez Human Gene ID | Enzyme | Description | Protein Function (Protein Atlas) |
|---|---|---|---|
| 64131 | XYLT1 | xylosyltransferase 1 | Potential drug targets; Enzymes; ENZYME proteins: Transferases; Disease related genes; Predicted secreted proteins |
| 64132 | XYLT2 | xylosyltransferase 2 | Predicted intracellular proteins; Potential drug targets; Enzymes; ENZYME proteins: Transferases; Disease related genes; Predicted secreted proteins |
| 11285 | B4GALT7 | beta-1,4-galactosyltransferase 7 | Predicted intracellular proteins; Potential drug targets; Enzymes; Disease related genes; ENZYME proteins: Transferases |
| 126792 | B3GALT6 | beta-1,3-galactosyltransferase 6 | Predicted intracellular proteins; Potential drug targets; Enzymes; Disease related genes; ENZYME proteins: Transferases |
| 26229 | B3GAT3 | beta-1,3-glucuronyltransferase 3 | Predicted intracellular proteins; Potential drug targets; Enzymes; Disease related genes; ENZYME proteins: Transferases |
| 55790 | CSGALNACT1 | chondroitin sulfate N-acetylgalactosaminyltransferase 1 | Enzymes; Predicted intracellular proteins; ENZYME proteins: Transferases |
| 55454 | CSGALNACT2 | chondroitin sulfate N-acetylgalactosaminyltransferase 2 | Enzymes; Predicted intracellular proteins; ENZYME proteins: Transferases |
| 337876 | CHSY3 | chondroitin sulfate synthase 3 | Enzymes; Predicted intracellular proteins; ENZYME proteins: Transferases |
| 22856 | CHSY1 | chondroitin sulfate synthase 1 | Potential drug targets; Enzymes; ENZYME proteins:Transferases; Disease related genes; Predicted secreted proteins |
| 79586 | CHPF | chondroitin polymerizing factor | Enzymes; Predicted intracellular proteins; ENZYME proteins: Transferases |
| 54480 | CHPF2 | chondroitin polymerizing factor 2 | Enzymes; Predicted intracellular proteins; ENZYME proteins: Transferases |
| 29940 | DSE | dermatan sulfate epimerase | Predicted intracellular proteins; Potential drug targets; ENZYME proteins: Isomerase; Enzymes; Disease related genes |
| 50515 | CHST11 | carbohydrate sulfotransferase 11 | Predicted intracellular proteins; Potential drug targets; Enzymes; Disease related genes; ENZYME proteins: Transferases |
| 55501 | CHST12 | carbohydrate sulfotransferase 12 | Enzymes; ENZYME proteins: Transferases |

| Entrez Human Gene ID | Enzyme | Description | Protein Function (Protein Atlas) |
|---|---|---|---|
| 166012 | CHST13 | carbohydrate sulfotransferase 13 | Enzymes; Predicted intracellular proteins; ENZYME proteins: Transferases |
| 9469 | CHST3 | carbohydrate sulfotransferase 3 | Enzymes; ENZYME proteins: Transferases; Disease related genes; Potential drug targets |
| 56548 | CHST7 | carbohydrate sulfotransferase 7 | Enzymes; Predicted intracellular proteins; ENZYME proteins: Transferases |
| 51363 | CHST15 | carbohydrate sulfotransferase 15 | Enzymes; Predicted intracellular proteins; ENZYME proteins: Transferases |
| 10090 | UST | uronyl 2-sulfotransferase | |
| 113189 | CHST14 | carbohydrate sulfotransferase 14 | Enzymes; ENZYME proteins: Transferases; Disease related genes; Potential drug targets |
